# A Novel Intersection: Cytomegalovirus Gastritis Following Cemiplimab and Talimogene Laherparepvec in a Patient With Advanced Cutaneous Squamous Cell Carcinoma

**DOI:** 10.1002/ccr3.9632

**Published:** 2024-12-15

**Authors:** Goar Egoryan, Alex Zimmet, Mingwei Yu, Joseph Pozdol, Aruna Subramanian, Sunil Reddy, Joanna Nelson

**Affiliations:** ^1^ Division of Oncology, Department of Medicine Stanford University School of Medicine Stanford California USA; ^2^ Department of Pathology Stanford University School of Medicine Stanford California USA; ^3^ Division of Infectious Diseases & Geographic Medicine, Department of Medicine Stanford University School of Medicine Stanford California USA

**Keywords:** cemiplimab, cytomegalovirus, immunotherapy, talimogene laherparepvec

## Abstract

Cytomegalovirus (CMV) reactivation is a rare complication in patients treated with immune checkpoint inhibitors (ICIs), typically occurring after immunosuppressive therapy for immune‐related adverse events (irAEs). Here, we report a unique case of severe CMV gastritis in a patient receiving cemiplimab, an anti‐PD‐1 antibody, and talimogene laherparepvec (T‐VEC), an oncolytic virus, without prior irAEs or immunosuppressive treatment. A 63‐year‐old man with advanced cutaneous squamous cell carcinoma received cemiplimab for one year and a single T‐VEC injection for recurrent disease. He presented with progressive dyspepsia, significant weight loss, and malnutrition requiring total parenteral nutrition. Endoscopy revealed extensive gastric ulceration, and biopsies confirmed CMV gastritis. Initial treatment with intravenous ganciclovir improved his viral load but provided minimal symptomatic relief. After six weeks of therapy, biopsies showed resolution of CMV infection, and the patient transitioned to oral valganciclovir for prophylaxis while resuming cancer treatment. This case highlights the potential for CMV reactivation in patients undergoing ICI therapy, even without prior immunosuppression or irAEs. Notably, the concurrent use of T‐VEC raises questions about the interplay between oncolytic viruses, ICIs, and immune modulation. Although T‐VEC is not known to directly cause CMV reactivation, its role in amplifying immune responses warrants further investigation. As ICIs and oncolytic viruses become increasingly integral in oncology, clinicians must remain vigilant for rare infectious complications like CMV reactivation. Further research is needed to elucidate mechanisms, identify risk factors, and optimize management strategies for these events.

**Summary**

- Cytomegalovirus may reactivate in cancer patients treated with immune checkpoint inhibitors, without prior immune events or immunosuppressive therapy, challenging existing views on cancer therapy, immune modulation, and infection risk.

## 1 Introduction

Immunotherapy has profoundly transformed the realm of oncology, now standing as a cornerstone in cancer treatment. At first, immune checkpoint inhibitors (ICIs) received approval solely for treating late‐stage or metastatic cancers, following or alongside chemotherapy. Today, however, their introduction at early cancer stages has cured many patients. While there have been remarkable advancements because of their efficacy, immune modulation can sometimes result in immune‐related adverse events (irAEs). Managing this often requires a spectrum of immunosuppressive drugs, from steroids to anti‐tumor necrosis factor‐alpha inhibitors. ICIs have also been tied to infectious complications, with limited data indicating a 7.3%–14% infection rate in solid tumor patients. Steroid and infliximab use for irAEs have emerged as significant risk factors for serious infections [1, 2].

Cytomegalovirus (CMV), a prevalent herpesvirus, typically leads to asymptomatic infections. Reactivation of a latent infection can occur during periods of immunosuppression, such as during treatment with corticosteroids, and may affect various organ systems. Surprisingly, despite ICIs' known impact on T‐cell function enhancement, there is scarce data associating ICI therapy with CMV reactivation—mostly following immunosuppressive therapy for irAEs [3], and less commonly, without previous immunosuppression [4].

Five percent of those diagnosed with cutaneous squamous cell carcinoma (SCC) have locally advanced or metastatic disease not suitable for definitive surgical or radiation approaches. In 2018, the Food and Drug Administration (FDA) approved cemiplimab (LIBTAYO, Regeneron Pharmaceuticals Inc.), an antibody against programmed cell death receptor‐1 (anti‐PD‐1), for such advanced skin SCC cases. Phase I/II clinical trials showed response rates between 42.9% and 50.8% for locally advanced and metastatic disease, and observational studies post‐approval documented rates of 32%–77% (median 58%) [5]. Another promising, yet non‐FDA‐approved treatment for advanced skin SCC is talimogene laherparepvec (T‐VEC), which is a modified herpes simplex virus, type 1 (HSV‐1). T‐VEC replicates within neoplastic cells, and accumulation of the virions leads to lysis of the cancer cells, causing necrosis, cell death, and the potential release of tumor‐associated antigens, priming anti‐tumor T‐cell responses. Notably, there is no documented literature on CMV reactivation following treatment with cemiplimab or T‐VEC.

Here, we present the case of a patient with locally advanced skin squamous cell carcinoma who, despite the absence of irAE‐gastritis or need for immunosuppressive therapy for irAE, developed severe CMV gastritis after treatment with the anti‐PD‐1 antibody cemiplimab and oncolytic virus (T‐VEC) therapy.

## 2 Case History/Examination

A 63‐year‐old South Asian man with a history of cutaneous SCC initially underwent superior gluteal cleft radiation therapy. After disease recurrence in the groin lymph nodes, he was treated with cemiplimab for 1 year, and, most recently, received one injection of T‐VEC into the groin lymph nodes. Following this, he was shortly hospitalized for non‐specific constitutional symptoms, received symptomatic therapy, and was discharged home with improvement. He returned to the hospital 2 weeks later, reporting dyspepsia of a 6‐month duration (progressively worsening in the past month), anorexia, and a 20 kg weight loss requiring initiation of total parenteral nutrition (TPN). The physical examination was unremarkable, except for epigastric tenderness.

## 3 Methods

**FIGURE 1:**
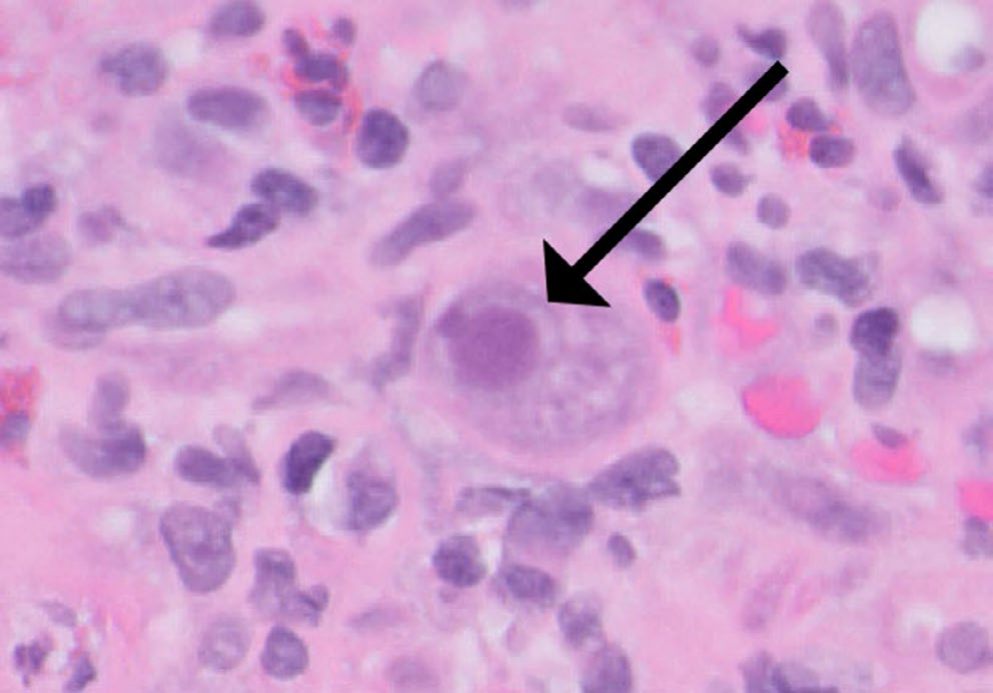
Stomach, antrum, biopsy, and H&E stain. H&E stain section of the gastric antrum with extensive ulceration, acute inflammation, and multiple CMV inclusions.

**FIGURE 2:**
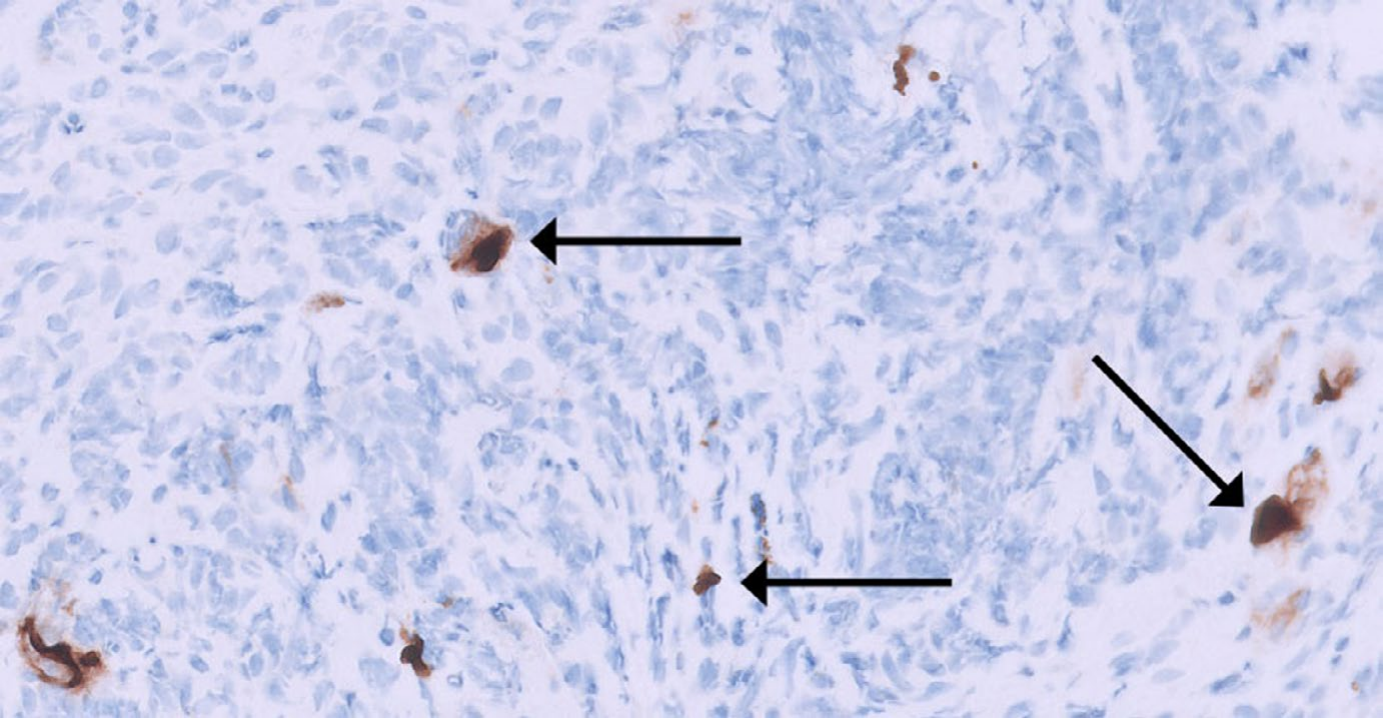
Stomach, antrum, biopsy, and CMV immunohistochemical preparation. Immunohistochemical staining for CMV highlights multiple CMV inclusions.

**FIGURE 3:**
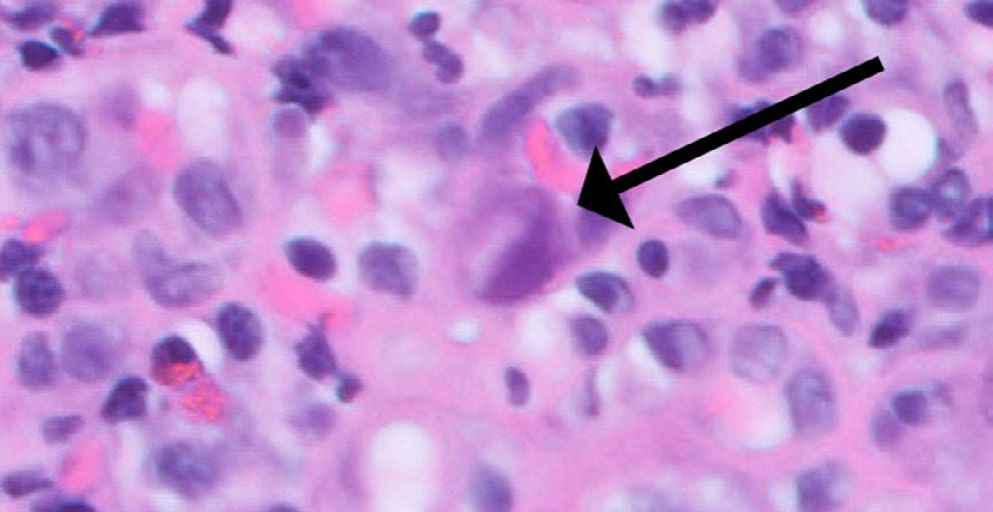
Stomach, biopsy, and H&E stain. H&E stain section of the gastric antrum with ulcerated mucosa and scattered eosinophilic nuclear and cytoplasmic inclusions.

**FIGURE 4:**
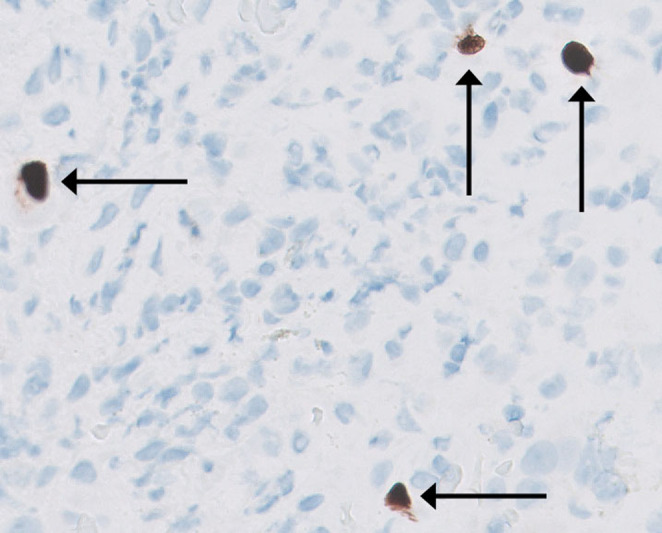
Stomach, biopsy, and CMV immunohistochemical preparation. Immunohistochemical staining for CMV highlights CMV inclusions.

Laboratory findings were notable only for hemoglobin (HGB) of 12 g/dL and albumin of 2.7 g/dL. The initial esophagogastroduodenoscopy (EGD) revealed extensive ulceration in the gastric antrum, and biopsy showed acute inflammation and multiple CMV inclusions (Figure 1) with positive CMV (Figure 2) and negative *Helicobacter pylori* (HP) immunohistochemistry; the duodenum was normal, both macro‐ and microscopically. A computed tomography (CT) scan of the abdomen and pelvis showed gastric wall thickening with mild enhancement of the gastric mucosa. The initial serum CMV viral load was 427 IU/mL. The patient was started on intravenous (IV) ganciclovir 5 mg/kg every 12 h. Given the rare association of CMV infection with ICI use, additional immunologic studies were sent but failed to identify any underlying immunodeficiency state that might have predisposed the patient to CMV infection. The total serum immunoglobulin G level was 541 mg/dL (normal range, 768–1632 mg/dL), the human immunodeficiency virus antigen/antibody screen test was negative, CD 4+ cell count was 335/μL (normal range, 300–1400/μL), CD 8+ cell count was 694/μL (normal range, 200–900/μL), CD 8+ TEMRA cell count was 453/μL (normal range, 25–280/μL), natural killer cell function was normal with an enumeration of 7% (normal range, 4%–25%), and T‐cell function was normal based on the tetanus‐induced lymphocyte proliferation test. Mild hypogammaglobulinemia was attributed to malnutrition, and elevated CD 8+ TEMRA cell count was linked to an active infection. Given the lack of clinical improvement, EGD was repeated on Day 7 of treatment and showed persistent ulcerated gastric mucosa, and biopsy showed intranuclear CMV inclusions (Figure 3) and positive CMV immunohistochemistry (Figure 4). On Day 11 of treatment, the serum CMV viral load had decreased to 135 IU/mL.

### 3.1 Outcome and Follow‐Up

Despite 3 weeks of IV antiviral therapy, the patient reported only minimal symptomatic relief. He was discharged home with a plan for continued IV ganciclovir and TPN. At Week 6 of IV ganciclovir, biopsies from the third EGD showed chronic active gastritis and peptic duodenitis with negative CMV and HP immunohistochemistry. After 7 weeks of IV ganciclovir, the patient reported significant improvement, with near‐normal oral intake. He was transitioned to valganciclovir (900 mg daily) for secondary prophylaxis in anticipation of upcoming cancer treatments. His TPN was discontinued 2 months after his discharge from the hospital.

## 4 Discussion

We present a case of a patient with advanced cutaneous SCC who developed severe CMV gastritis after receiving cemiplimab, an anti‐PD‐1 antibody, and T‐VEC oncolytic virus therapy.

While CMV infection in individuals with a healthy immune system often shows no symptoms or appears like a viral or mononucleosis‐type illness [6, 7], it can also manifest in the gastrointestinal, neurological, hematological, skin, cardiac, or ocular regions, primarily as a reactivation [7, 8]. CMV gastritis is an uncommon and frequently overlooked condition, typically seen alongside widespread CMV infection. The frequency of CMV infections in the upper digestive system remains undefined [9].

The development of invasive CMV disease in a host like our patient was an unexpected event, given that ICIs work by enhancing T‐cell immunity, which is vital for anti‐viral protection. The precise rate at which CMV reactivates in patients undergoing ICI treatment remains unclear but is believed to be minimal. Tay et al. [3] noted a mere 0.3% overall occurrence of CMV detection in those administered ICIs. In a vast majority of cases, invasive CMV disease in patients with solid malignancies on ICIs complicated irAEs that required high‐dose corticosteroids and other immunomodulatory drugs [3, 10, 11, 12, 13, 14, 15, 16, 17, 18, 19, 20], which could be explained by the fact that CMV has tropism to inflamed tissues. Consequently, there is a significant occurrence of CMV infection among patients with severe acute inflammatory bowel disease (IBD), and the frequency is even higher in those with steroid‐resistant conditions [21].

Interestingly, our patient developed invasive CMV disease without pre‐existing inflammation in the form of irAE‐gastritis, which is typically treated with high doses of corticosteroids. Furthermore, there was no concurrent *H. pylori* infection or a history of current immunosuppression, as reported in the literature [3, 4, 10, 11, 12, 13, 14, 15, 16, 17, 18, 19, 20]. However, our patient complained of dyspepsia for 6 months halfway through his cemiplimab therapy course, which may have represented an unrecognized form of irAE‐gastritis before he was diagnosed with CMV.

While the histologic features of ICI‐related colitis have been widely reported, there is less data on the upper gastrointestinal (GI) manifestations of irAE. The morphological spectrum of ICI therapy‐associated gastritis typically involves diffuse chronic active gastritis, characterized by increased intraepithelial lymphocytes and prominent apoptosis. However, in a subset of patients, it can present as a focal enhancing gastritis with granulomatous inflammation, reminiscent of the gastric involvement seen in Crohn's disease [22]. In another study, 39% of patients were also found to have gastric peri‐glandular inflammation [23]. While our patient's upper GI biopsies did not show features typically associated with ICI‐associated irAE‐gastritis, the classic morphology of invasive CMV infection was present. Symptom improvement with antiviral therapy without steroids also supported the infectious process.

Cases of CMV reactivation in patients on ICIs and not previously treated with immunosuppressants are rare [4, 24, 25, 26]. In three of those cases [4, 24, 25], CMV gastritis developed without definitive evidence of pre‐existing irAE‐gastritis, like in our patient, but all patients received either pembrolizumab or atezolizumab and not cemiplimab.

Another aspect of our case that has not been previously described in conjunction with CMV infection is T‐VEC therapy. T‐VEC is a live attenuated genetically modified HSV designed for replication in tumor cells and local expression of granulocyte‐macrophage colony‐stimulating factor (GM‐CSF) by the infected tumor cells, which is proposed to enhance tumor antigen presentation to the immune system, inducing immune responses to the tumors, but the exact mechanism of action is not known [27, 28, 29]. At present, T‐VEC stands as the sole FDA‐approved viral therapy for melanoma [30, 31]. Nonetheless, it has demonstrated efficacy both as a standalone treatment and when combined with other therapies for cancers other than melanoma [32, 33, 34, 35, 36, 37, 38]. According to the official FDA report, the most common treatment‐emergent adverse events (≥ 25%) included fatigue, chills, pyrexia, nausea, influenza‐like illness, and injection site pain. Local and systemic infections following T‐VEC injection have been reported, but the exact incidence is unknown. In one study, 12/52 (23%) patients had any microbiologically confirmed infection identified following T‐VEC therapy: 6 were bacterial (3 urinary tract infection, 1 bloodstream, and 2 wounds), and 9 were viral (5 respiratory, 3 GI, and 1 localized HSV infection with dermal lesions more than 1 year after the final T‐VEC) [39]. A disseminated herpes infection following T‐VEC injection has been reported followed by prolonged melanoma control without further therapy [40]. Upon review of the literature and the results of 23 ongoing T‐VEC trials, only one case of invasive CMV disease (colitis) was found in a patient who had received T‐VEC, but in combination with ipilimumab, and no further details were available [41]. Our patient had already been experiencing dyspepsia for 5 months prior to T‐VEC therapy but reported significant symptom worsening shortly after receiving the oncolytic viral treatment. The role of T‐VEC therapy in this context, although utilizing a CMV promoter, seems unlikely to be the direct culprit behind CMV gastritis because the CMV promoter does not carry genes responsible for infectivity and latency [28]. However, it is theoretically possible that the use of T‐VEC might have triggered an immune reaction to CMV in the stomach, potentially resembling an abscopal phenomenon. This phenomenon refers to tumor regression in both the targeted lesion and any untreated tumors at a distant site, which can be caused by local radiation therapy, with or without systemic immunotherapy [42, 43]. The complex interplay between T‐VEC, ICIs, and the immune system is still unknown and merits further investigation.

To the best of our knowledge, this is the first reported case of CMV gastritis in a patient treated with both cemiplimab and T‐VEC. While CMV reactivation in patients not previously exposed to immunosuppressants is infrequent, the manifestation in our subject was particularly intriguing given the absence of an inflammatory pattern typically associated with such cases. This report heightens awareness of the potential complications associated with ICI treatments and T‐VEC. As the medical community ventures deeper into the realm of immunotherapies, both local and systemic, it is important to understand the mechanism of infectious complications, identify potential biomarkers or risk factors associated with the development of those complications, and establish proper surveillance.

## Author Contributions

**Goar Egoryan:** visualization, writing – original draft, writing – review and editing. **Alex Zimmet:** writing – review and editing. **Mingwei Yu:** writing – review and editing. **Joseph Pozdol:** writing – review and editing. **Aruna Subramanian:** supervision, writing – review and editing. **Sunil Reddy:** supervision, writing – review and editing. **Joanna Nelson:** supervision, visualization, writing – review and editing.

## Consent

Written informed consent was obtained from the patient to publish this report in accordance with the journal's patient consent policy.

## Conflicts of Interest

The authors declare no conflicts of interest.

## Data Availability

Data sharing is not applicable to this article as no datasets were generated or analyzed during the current study.
